# Three-year results of the MOTION randomized controlled trial for treatment of lumbar spinal stenosis using the percutaneous *mild*® Procedure

**DOI:** 10.1016/j.inpm.2025.100561

**Published:** 2025-03-12

**Authors:** Shrif J. Costandi, Timothy R. Deer, Timothy B. Chafin, Christopher Kim

**Affiliations:** a Cleveland Clinic, 9500 Euclid Ave, Cleveland, OH, 44195, USA; b The Spine & Nerve Centers of the Virginias, 400 Court Street, Suite 100, Charleston, WV, 25301, USA; c Vidant Roanoke-Chowan Hospital, 500 S. Academy St, Ahoskie, NC, 27910, USA

**Keywords:** *mild*, Hypertrophic ligamentum flavum, (HLF), Neurogenic claudication, Lumbar spinal stenosis, (LSS), Low back pain, Percutaneous image-guided lumbar decompression, (PILD)

## Abstract

**Objective:**

The MOTION prospective, multicenter randomized controlled trial compares the outcomes of percutaneous image-guided lumbar decompression in combination with conventional medical management (CMM) to the use of CMM alone for the treatment of lumbar spinal stenosis with neurogenic claudication secondary to hypertrophic ligamentum flavum. The study includes extended follow-up for patients in both the treatment group and for those who crossed over from the control group to the treatment group.

**Methods:**

The treatment group received the *mild*® Procedure (Vertos Medical, Aliso Viejo, CA, USA) in combination with nonsurgical CMM, while the active control group received CMM alone. There were no restrictions for either group regarding access to real-world CMM therapies. Patients reported outcomes using the Oswestry Disability Index (ODI), Zurich Claudication Questionnaire (ZCQ), and Numeric Pain Rating Scale (NPRS). Objective outcomes were measured using a validated Walking Tolerance Test (WTT), the incidence of subsequent lumbar spine interventions, and the occurrence of adverse events.

**Results:**

Forty-eight patients initially receiving *mild* + CMM consented to extended follow-up and were available for 3-year follow-up. All outcomes for this group were significantly improved over baseline (p-values ranging from <0.0001 to 0.0001). At 3-year, ODI, NPRS back and leg, ZCQ symptom severity, and physical function improved by 16.9, 3.0, 4.3, 0.8, and 0.6, respectively. Walking tolerance test demonstrated 274 % improvement from baseline, and only 4 (5.6 %) patients had received surgical intervention. No device- or procedure-related adverse events were reported.

**Conclusions:**

MOTION 3-year follow-up results continue to demonstrate the safety and durability of the *mild* Procedure combined with CMM for early interventional treatment of symptomatic LSS. The absence of device or procedure-related adverse events further underscores the robust safety profile of the *mild* Procedure. Significant and substantial improvements in all the outcomes were observed from baseline to follow-up for patients treated with the *mild* Procedure. These results support the *mild* Procedure as an effective approach for early intervention in LSS treatment.

## 1 Introduction

Lumbar spinal stenosis (LSS), most often associated with advancing age, results from the natural progression of degenerative changes of the lower spine resulting in a narrowing of the spinal canal [1,2]. While these changes can include loss of intervertebral disc height and hypertrophy of the facet joints, hypertrophy of the ligamentum flavum can also result in narrowing of the canal, especially during activities that increase spinal loading and extension such as walking upright. This narrowing often results in back pain, radicular pain and neurogenic claudication [3,4]. The effect of neurogenic claudication on mobility can be debilitating and can strongly impact health-related quality of life, resulting in a more sedentary lifestyle [[5], [6], [7]].

First-line treatments for LSS have historically focused on conservative, non-surgical options such as medications, spinal injections, physiotherapy and lifestyle modifications [7]. Surgery has, until recently, been the next step in the treatment continuum for those unresponsive to conservative therapies and includes direct decompression of the neural structures by open, minimally invasive or endoscopic surgical approaches and indirect decompression through use of interspinous spacers [[7], [8], [9]]. A percutaneous image-guided approach for lumbar decompression (PILD) was introduced over a decade ago and has been shown to be superior to conventional medical management (CMM) and when compared to surgical interventions whether open or indirect spacers, *mild* had a favorable safety profile with less incidence complications [[10], [11], [12], [13], [14]]. The utility of the PILD approach when used earlier in the treatment continuum, either as soon as LSS is diagnosed or after failure of the first epidural spinal injection, has also been shown [15].

The MOTION study, a randomized controlled trial, compares the effectiveness of the *mild* Procedure in combination with CMM (*mild* + CMM) in treating LSS in patients with neurogenic claudication to the use of CMM alone (CMM-Alone). Results from the MOTION study at 6-month, 1-year and 2-year follow-ups have previously been reported [11,16,17]. Per the MOTION study protocol, patients in the *mild* + CMM group, as well as any CMM-Alone patients who crossed over to receive *mild,* will continue to be evaluated annually through 5-year extended follow-up. This report presents the results of the 3-year follow-up for MOTION study participants who initially received the *mild* + CMM treatment and consented to the extended follow-up period.

## 2 Methods

Informed patient consent for the MOTION study was obtained prior to study enrollment, as was Institutional Review Board approval. In addition, participants in the 3 to 5-year follow-up who were available for their visit, considered as a modified intent-to-treat (mITT) population, provided explicit consent for this extended time period. All analyses were performed with this mITT group. The study was conducted in accordance with the International Conference on Harmonization guidelines for Good Clinical Practices and was registered as NCT03610737 with the Clinical Trial Registry (clinicaltrials.gov).

### 2.1 Patients

Patient eligibility was determined using selection criteria that included age of 50–80, neurogenic claudication symptoms for at least 3 months, and assessment of magnetic resonance or computed tomographic images by an independent medical monitor. The complete inclusion/exclusion criteria have been previously reported [11]. Patients selected for inclusion were randomly assigned to the *mild* Procedure combined with CMM (*mild* + CMM) treatment group or to CMM without the use of *mild* (CMM-Alone) control group in a 1:1 ratio. Patients in the CMM-Alone group were allowed to cross over to the *mild* + CMM group after 1-year follow-up. Patients in the *mild* + CMM group who signed a consent for extended follow-up of 3–5 years are included in this 3-year report.

#### 2.1.1 Conventional medical management

Conventional medical management consisted of therapies commonly used as first-line treatments for LSS with neurogenic claudication, including oral pain medications, home exercise, physical therapy, lumbar injections such as epidural steroid injections. Each investigator determined the CMM regimen for individual study participants based on their specific needs, symptoms, and medical background.

#### 2.1.2 The *mild* Procedure

Following application of local anesthetic and light sedation, the *mild* Procedure accesses the posterior lumbar spine through a single 5.1 mm incision for placement of a stabilizing portal under fluoroscopic imaging. Specialized surgical tools are passed through the portal to remove portions of the lamina and debulk the ligamentum flavum, thereby decompressing the underlying neural tissues. A detailed description of the *mild* Procedure has been previously reported [14].

### 2.2 Outcome measures

Patient-reported outcome tools were used to subjectively measure performance outcomes of the *mild* Procedure at all follow-up periods. The Oswestry Disability Index (ODI) was used to evaluate pain and functional disability as the primary study outcome. The Numeric Pain Rating Scale (NPRS) measuring back and leg pain and Zurich Claudication Questionnaire (ZCQ) assessing symptom severity and physical function specific to LSS were both used as secondary study outcomes.

Objective performance measures were also included as secondary study outcomes. In a validated Walking Tolerance Test (WTT), patients walked at their own pace up to 15 min, or until they began to experience severe symptoms of neurogenic claudication [18]. The rate of subsequent lumbar spine intervention (SLSI) sought by study patients was also recorded. Any device-related and procedure-related adverse events (AEs) and serious adverse events (SAEs) were recorded and adjudicated by an independent clinical event monitor.

### 2.3 Analysis methods

Continuous data are summarized using means and standard deviations, while summaries of categorical variables are represented by frequency counts and percentages. For patient experiencing multiple events, percentages are calculated based on both the number of patients who experienced the event and the total number of events they encountered. Comparisons of means used a two-tailed *t*-test with a significance level of 0.05. Paired t-tests were used for within-group analyses using dependent samples. Between group analysis employed t-tests assuming unequal sample variances. Fisher's exact test was used for frequency analyses. VassarStats (http://www.vassarstats.net) was used for all analyses.

## 3 Results

### 3.1 Patient characteristics

**Fig. 1:**
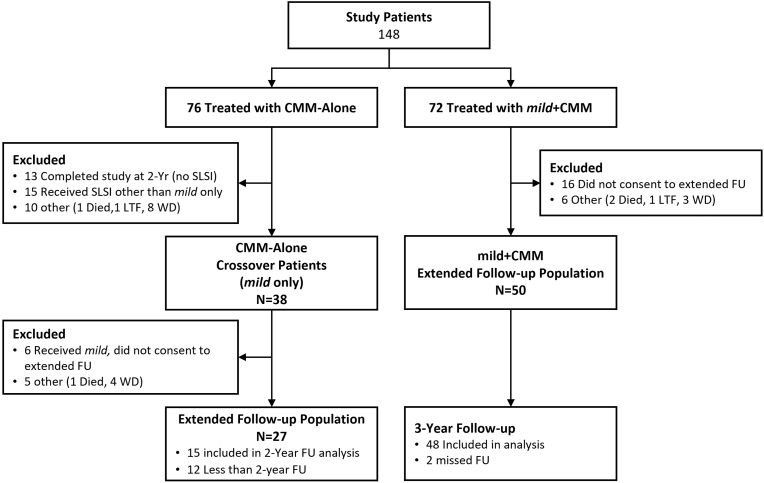
Extended follow-up study patient flow. SLSI = subsequent lumbar spine intervention, FU = follow-up, LTF = Lost to follow-up, WD=Withdrew.

The study was conducted at 19 institutions in the U.S., with 77 patients assigned to the *mild* + CMM group and 78 patients to the CMM-Alone group. Seventy-two patients in *mild* + CMM and 76 in CMM-Alone received treatment from September 2018 through December 2019. Fifty of 72 patients treated in the *mild* + CMM group consented to extended follow-up to include 3, 4 and 5 years. Two patients in this group missed the 3-year follow-up. The modified intent-to-treat population for the *mild* + CMM group consists of the remaining 48 mild + CMM patients (Fig. 1). Thirty-eight patients in the CMM-Alone group crossed over elected to cross over to receive the *mild* Procedure, and 27 of these patients consented to the extended follow-up.

Demographic and baseline outcome metrics of these groups have been previously reported [11]. The mean age for patients in the *mild* + CMM group was 64.7 years and 66.8 years for patients in the CMM-Alone group. Male to female ratio was equally distributed with 57.7 % in the *mild* + CMM group and 57.1 % for the CMM-Alone group being female. There were no significant variations in demographic or baseline measures between the groups.

### 3.2 Outcome measures

**Fig. 2:**
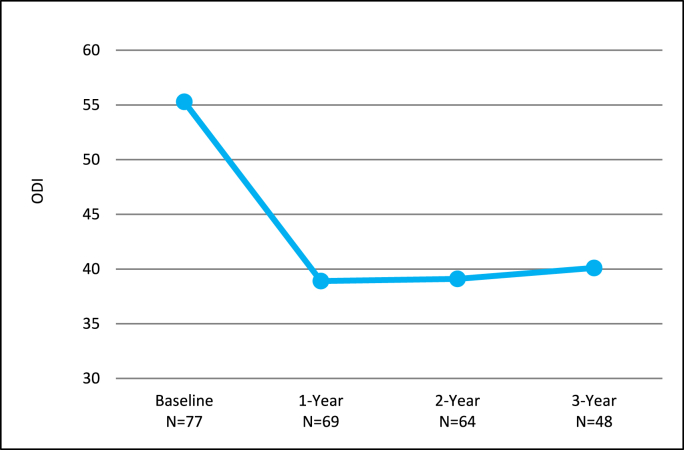
Oswestry Disability Index (ODI) mean outcomes for the *mild* + CMM group.

**Fig. 3:**
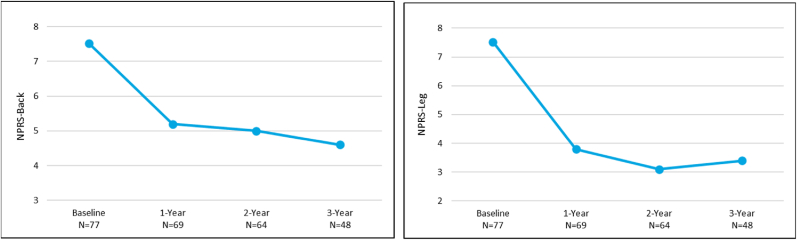
Numeric Pain Rating Scale (NPRS) mean outcomes for back and leg pain for the *mild* + CMM group.

**Fig. 4:**
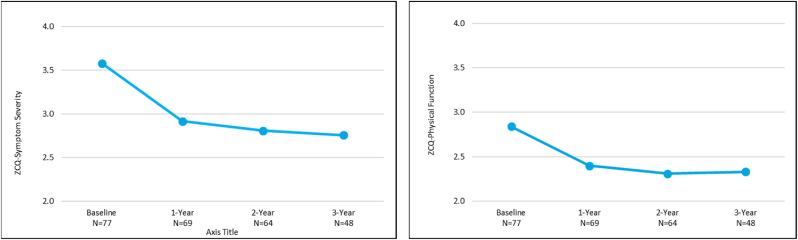
Zurich Claudication Questionnaire (ZCQ) mean outcomes for symptom severity and physical function for the mild + CMM group.

At 3 years, the mean ODI was significantly improved by 16.9 points (p < 0.0001) (Fig. 2). Patients also reported significant improvements of 3.0 and 4.3 points in mean back and leg pain NPRS scores, respectively (p < 0.0001) (Fig. 3). The mean ZCQ scores for symptom severity and physical function at 3 years were significantly improved by 0.8 and 0.6 points, respectively, compared to baseline (p < 0.0001, p = 0.0001) (Fig. 4).

**Fig. 5:**
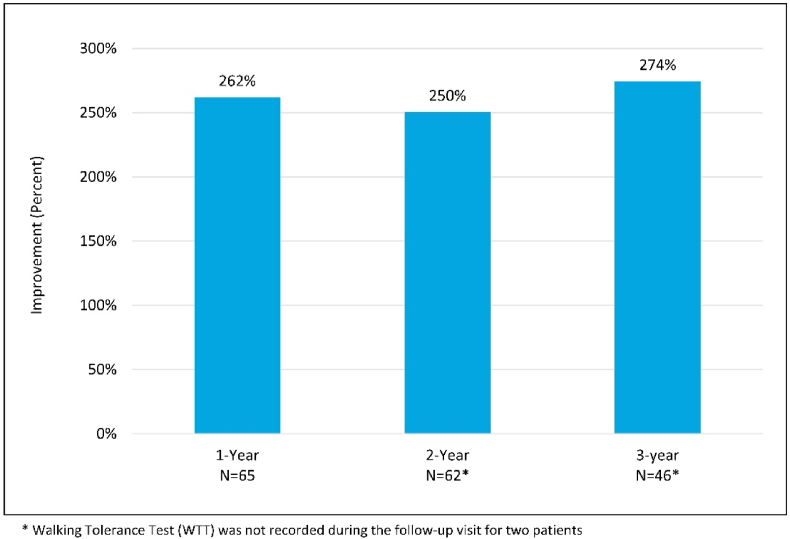
15-Minute Walking Tolerance Test mean percent improvement for the *mild* + CMM group.

Walking times using the objective Walking Tolerance Test significantly improved by a mean of 274 % at 3-year follow-up when compared to baseline (p < 0.0001) (Fig. 5), ranging from 0 min to the full 15-min limit of the test.

Of the 48 patients available for extended follow-up in this report, 8 patients underwent an SLSI (5 occurred at the level of initial *mild* treatment). Of these, two patients were in the first year of follow-up (1 laminectomy and 1 non-specific lumbar decompression), three patients were between 1 and 2Y follow-up (1 neurostimulator, 1 fusion, and 1 adhesiolysis), and three patients were between 2Y and 3Y follow-up (1 interspinous spacer, 1 fusion, and 1 *mild*). No device- or procedure-related adverse events of any kind were reported for patients in the *mild* + CMM group through the 3-year follow-up. Three additional unrelated serious adverse events (SAEs) in three patients were reported between 2- and 3-year follow-up. The three events consisted of an aortic aneurysm dissection, a cardiac arrest, and a broken tibia and fibula, two of which resulted in death.

## 4 Discussion

The percutaneous image-guided approach for lumbar decompression, currently performed only with the *mild* Procedure, has become a recognized treatment for LSS secondary to a hypertrophied ligamentum flavum. In a 2022 summary of best practices for the minimally invasive treatment of lumbar spinal stenosis (MIST 2.0), the American Society of Pain and Neuroscience (ASPN) concluded that “PILD should be considered for the treatment of mild-to-moderate LSS in the presence of NC, with less than or equal to a grade 2 spondylolisthesis, and with a contribution of spinal narrowing with at least 2.5 mm of LFH.” Based on the mounting evidence, societal guidelines recommend mild as first line therapy [19]. Similarly, the Pacific Spine and Pain Society recognizes Level I and II evidence that suggests improvements in pain and function scores can be achieved in patients indicated for PILD treatment [20].

Patient safety of this approach has been compared to the use of interspinous spacers and outpatient laminectomy for the treatment of LSS using Medicare claims data. When evaluated over two years from the date of treatment, the rate of harms for the interspinous spacer was more than twice that of the *mild* Procedure [12]. Patients experienced harms more than three times as often when treated with outpatient laminectomy versus *mild* when evaluated over two years [13]. These differences were significant for both investigations (p < 0.0001). In the present study, the safety of the *mild* Procedure was reinforced by the continued lack of any device- or procedure-related adverse events through the 3-year follow-up.

While numerous studies have reported 1- and 2-year functional and pain outcomes for patients treated with the *mild* Procedure [16,[21], [22], [23], [24]]^,^ this is the first prospective real world study to present longer-term results with these measures. Patients in the *mild* + CMM group in the present randomized controlled trial experienced significant improvements in both objective walking tolerance testing and subjective self-reported outcome measures at 6-month, 1-year and 2-year follow-up visits [11,16,17], and these significant improvements were still evident at 3-year follow-up.

The improvements in ODI, NPRS, and ZCQ at the 1 and 2 year follow-up visits compared to baseline in the mild + CMM group continued when assessed at 3 years (Fig. 2, Fig. 3, Fig. 4). The substantial improvement in the objective Walking Tolerance Test results seen at 1 and 2 years post-treatment was maintained for the *mild* + CMM patients available for 3-year follow-up. The improvements in all of the outcome measure results were statistically significant when compared to baseline (Table 1).

**Table 1:** Outcome measure comparison between 1, 2, and 3 Year follow-up visits.

Outcome Measure Improvement (Mean ± SD) P-value (within group)	1-Year N = 69	2-Year N = 64	3-Year N = 48
ODI	16.5 ± 19.3	17.0 ± 21.4	16.9 ± 20.5
p-value	<0.001	<0.0001	<0.0001

NPRS			
Back	2.4 ± 2.8	2.6 ± 3.0	3.0 ± 2.8
p-value	<0.0001	<0.0001	<0.0001

Leg	3.8 ± 3.1	4.5 ± 3.0	4.3 ± 2.9
p-value	<0.0001	<0.0001	<0.0001

ZCQ			
Symptom Severity	0.7 ± 0.8	0.8 ± 1.0	0.8 ± 1.0
p-value	<0.0001	<0.0001	<0.0001

Physical Function	0.5 ± 0.7	0.6 ± 0.8	0.6 ± 0.7
p-value	<0.0001	<0.0001	<0.0001

Walking Tolerance Test	262 %	250 %	274 %
p-value	<0.0001	<0.0001	0.0021

By 3-year follow-up, 10 of the 72 patients who received treatment in the *mild* + CMM group underwent some type of a SLSI. For 4 of these patients the SLSI was an open decompression, fusion, or laminectomy, representing a rate of 5.6 % of the *mild* + CMM group treated, which is comparable with the results of other studies of the *mild* Procedure. Rates of subsequent lumbar spine surgeries have been reported by Chopko et al. as 5.2 % of 58 patients within two years following an initial *mild* treatment [24] and by Mekhail et al. as 12 % of 75 patients within five years of treatment [25]. A retrospective US claims database review by Shahzad et al. of 3222 patients having received the *mild* Procedure from January 2010 through October 2021 found a subsequent rate of surgical decompression/laminectomy of 8.8 % within 5 years of treatment with the *mild* Procedure [26].

Patients in the CMM-Alone group were unrestricted in the conventional therapies they could receive (except for *mild*, of course), and included exercise, physical therapy, and lumbar injections [16]. Failure of CMM to alleviate symptoms in this group was reported as 69.7 % (53/76), with 38 patients crossing over to be treated with the *mild* Procedure. The rate of CMM failure and crossover to the *mild* group in the present study is consistent with conventional treatment failure and crossover rates in other studies of LSS treatments [[27], [28], [29], [30], [31], [32], [33]].

A supplementary analysis was conducted to evaluate 2-year outcomes for patients who were originally in the CMM-Alone study group, and who then crossed over to receive the *mild* Procedure only. This group included the 15 study patients who consented to extended follow-up and reached the 2-year follow-up timepoint. The mean time to crossover for these 15 patients was 11.5 months (range: 1.1–16.8 months) before receiving the *mild* Procedure. At two years, within group improvements were statistically significant for all outcome measures (p-value ranging from <0.0001 to 0.03). The ODI improved by 14.3 points (p = 0.03), NPRS back and leg improved by 3.1 and 4.7 points (p = 0.0001, p < 0.0001), respectively, and ZCQ symptom severity and physical function improved by 0.8 and 0.5 points (p = 0.0028, p = 0.03), respectively. The WTT showed 209 % improvement from baseline (p = 0.0033). These results are comparable to the 2 year follow-up of the original *mild* + CMM cohort [17].

While the MOTION study reflects the real-world clinical environment of LSS treatment, the diversity of CMM treatment options and algorithms may be a limitation. However, patients in both groups were allowed the freedom to receive the desired CMM therapies throughout the study. Another limitation is the loss of specific treatment performance in the CMM-Alone group due to the high rate of crossover. Consequently, this provided a valuable opportunity to review this subset group that received extended CMM therapy before the *mild* Procedure. However, due to the varying timing of the crossover, the number of patients reaching a meaningful follow-up is currently limited. The strength of the results for the crossover group may improve in future analyses as more patients reach 2-year follow-up.

## 5 Conclusion

The ability of the *mild* Procedure to safely and effectively treat LSS with neurogenic claudication secondary to hypertrophic ligamentum flavum continued through the three-year follow-up in the MOTION study, demonstrating the durability of percutaneous decompression as first-line treatment. The *mild* Procedure continues to demonstrate a reduction in SLSIs, including surgery. The absence of any device- or procedure-related adverse events reported in this study confirms the safety profile of the *mild* Procedure. Analysis of the crossover patients provided an opportunity to evaluate the effectiveness of the *mild* Procedure following an extended course of conventional medical management. Crossover patient outcomes at two years following *mild* treatment were statistically significantly improved versus baseline.

## Disclosures

Shrif Costandi has received research funding from Vertos, Vivex, Mainstay and Saluda (Paid to the Cleveland Clinic). Timothy Deer is the research principal investigator on this study and is a consultant for Abbott, Saluda, CornerLoc, PainTEQ, Boston Scientific (Relivant), Spinal Simplicity, Aurora, and Nervonik. He has received research funding from Abbott, Boston Scientific, Vertos, Saluda, PainTEQ, Saluda. Tim Chafin has received research funding from Vertos. Christopher Kim is a consultant for Boston Scientific (Vertiflex), Vertos, PainTEQ, and Abbott. The authors report no other conflicts of interest in this work.
